# Supplementary material for: Modelling Skylarks (Alauda arvensis) to Predict Impacts of Changes in Land Management and Policy: Development and Testing of an Agent-Based Model
Source: PLoS One. 2013 Jun 6;8(6):e65803. doi: 10.1371/journal.pone.0065803 (PMC3675089; doi:10.1371/journal.pone.0065803)
Supplement: Supporting Information S4 — The skylark ODdox as a zipped archive. (ZIP) [file pone.0065803.s004.zip › Skylark_ODdox/class_weather-members.html]

ALMaSS Skylark ODdox: Member List


ALMaSS Skylark ODdox
 2.0


- Main Page
- Related Pages
- Classes
- Files

- Class List
- Class Index
- Class Hierarchy
- Class Members

Weather Member List

This is the complete list of members for Weather, including all inherited members.

|  |  |  |
| --- | --- | --- |
| GetDDDegs(long a\_date) | Weather |  |
| GetGlobalRadiation(long a\_date) | Weather | inline |
| GetGlobalRadiation(void) | Weather | inline |
| GetMeanTemp(long a\_date, unsigned int a\_period) | Weather |  |
| GetRain(long a\_date) | Weather | inline |
| GetRain(void) | Weather | inline |
| GetRainPeriod(long a\_date, unsigned int a\_period) | Weather |  |
| GetSnow(long a\_date) | Weather |  |
| GetSnow(void) | Weather | inline |
| GetTemp(long a\_date) | Weather | inline |
| GetTemp(void) | Weather | inline |
| GetTempPeriod(long a\_date, unsigned int a\_period) | Weather |  |
| GetWind(long a\_date) | Weather | inline |
| GetWind(void) | Weather | inline |
| GetWindPeriod(long a\_date, unsigned int a\_period) | Weather |  |
| m\_datemodulus | Weather | private |
| m\_insolation | Weather | private |
| m\_rain | Weather | private |
| m\_rainingtoday | Weather | private |
| m\_raintoday | Weather | private |
| m\_snow\_decay | Weather | private |
| m\_snowtoday | Weather | private |
| m\_temp | Weather | private |
| m\_temptoday | Weather | private |
| m\_wind | Weather | private |
| m\_wind\_valid | Weather | private |
| m\_winddir | Weather | private |
| m\_winddir\_valid | Weather | private |
| m\_winddirtoday | Weather | private |
| m\_windtoday | Weather | private |
| Raining(void) | Weather | inline |
| Tick(void) | Weather |  |
| Weather(const char \*a\_weatherdatafile) | Weather |  |
| ~Weather(void) | Weather |  |


- Generated on Thu Jan 10 2013 13:15:38 for ALMaSS Skylark ODdox by
   1.8.1.1
